# Modeling Soil Organic Carbon at Regional Scale by Combining Multi-Spectral Images with Laboratory Spectra

**DOI:** 10.1371/journal.pone.0142295

**Published:** 2015-11-10

**Authors:** Yi Peng, Xiong Xiong, Kabindra Adhikari, Maria Knadel, Sabine Grunwald, Mogens Humlekrog Greve

**Affiliations:** 1 Department of Agroecology, Faculty of Science and Technology, Aarhus University, Blichers Allé 20, P.O. Box 50, DK-8830 Tjele, Denmark; 2 Department of Soil and Water Science, University of Florida, Gainesville, FL 32611, United States of America; 3 Department of Soil Science, FD Hole Soils Lab, University of Wisconsin−Madison, Madison, WI 53706, United States of America; DOE Pacific Northwest National Laboratory, UNITED STATES

## Abstract

There is a great challenge in combining soil proximal spectra and remote sensing spectra to improve the accuracy of soil organic carbon (SOC) models. This is primarily because mixing of spectral data from different sources and technologies to improve soil models is still in its infancy. The first objective of this study was to integrate information of SOC derived from visible near-infrared reflectance (Vis-NIR) spectra in the laboratory with remote sensing (RS) images to improve predictions of topsoil SOC in the Skjern river catchment, Denmark. The second objective was to improve SOC prediction results by separately modeling uplands and wetlands. A total of 328 topsoil samples were collected and analyzed for SOC. Satellite Pour l’Observation de la Terre (SPOT5), Landsat Data Continuity Mission (Landsat 8) images, laboratory Vis-NIR and other ancillary environmental data including terrain parameters and soil maps were compiled to predict topsoil SOC using Cubist regression and Bayesian kriging. The results showed that the model developed from RS data, ancillary environmental data and laboratory spectral data yielded a lower root mean square error (RMSE) (2.8%) and higher R^2^ (0.59) than the model developed from only RS data and ancillary environmental data (RMSE: 3.6%, R^2^: 0.46). Plant-available water (PAW) was the most important predictor for all the models because of its close relationship with soil organic matter content. Moreover, vegetation indices, such as the Normalized Difference Vegetation Index (NDVI) and Enhanced Vegetation Index (EVI), were very important predictors in SOC spatial models. Furthermore, the ‘upland model’ was able to more accurately predict SOC compared with the ‘upland & wetland model’. However, the separately calibrated ‘upland and wetland model’ did not improve the prediction accuracy for wetland sites, since it was not possible to adequately discriminate the vegetation in the RS summer images. We conclude that laboratory Vis-NIR spectroscopy adds critical information that significantly improves the prediction accuracy of SOC compared to using RS data alone. We recommend the incorporation of laboratory spectra with RS data and other environmental data to improve soil spatial modeling and digital soil mapping (DSM).

## Introduction

Reliable information on the spatial distribution of soil physical and chemical properties is required for sustainable land management and precision agriculture [1, 2]. During the past 20 years, passive remote sensing (RS) has been widely applied to digital soil mapping (DSM) since it overcomes the issue of a shortage of soil data at regional and national scale [3–5]. Grunwald, Thompson [6] proposed the STEP-AWBH model (S, soil; T, topography; E, ecology; P, parent material; A, atmosphere; W, water; B, biotic; H, human) to determine soil properties and classes. Based on this model, they declared that the “B” factor representing vegetation cover, land use and other ecophysiological biotic properties could be assessed through spectral indices derived from passive remote sensing imagery. These data can be easily acquired from agencies, for instance the Satellite Pour l’Observation de la Terre (SPOT) and Landsat Data Continuity Mission images. Numerous studies have derived different vegetation indices from imagery to indirectly obtain information on soil properties [3, 7]. These techniques are useful for mapping large areas since it reduces the need for costly soil sampling and laboratory analyses.

Mulder, de Bruin [3] comprehensively reviewed the link between variation of vegetation cover and soil biogeochemical properties from which a series of vegetation indices based on the RS imagery can be used for modeling soil properties and DSM. One of the most common indicators of the live green vegetation is the Normalized Difference Vegetation Index (NDVI) [8]. The NDVI is mainly an indicator of plant vigor as it uses the characteristic “red edge” feature of plant spectra [7]. In order to extract sufficient information from RS imagery for the prediction of soil properties, several indices have been proposed besides the NDVI. Kim., Grunwald [9] used nine different spectral vegetation indices derived from three different satellite images and environmental ancillary data, and developed prediction models for soil phosphorus (TP) and total nitrogen (TN) for a wetland area. They found that the TVI[10], SR[11], and NDVIgreen[12] were the three most important predictors for TP. The results also showed that the NDVI index did not contribute strongly to the prediction ability in the complex wetland ecosystem. The main drawbacks of multispectral sensor data are the large pixel size and the wide spectral bands from which it is difficult to capture detailed spectral information for soil [4, 13]. Thus, spectral indices derived from remote-sensing imagery might not provide sufficient information for determining soil properties [2, 3]. Combining RS images with a high-spectral-resolution data source (i.e., laboratory spectroscopy) for modeling soil properties would therefore provide a promising solution.

In the last 15 years, Vis-NIR spectroscopy has been widely adapted for the analysis of soil properties under laboratory conditions [14]. This technique has been commonly considered a potentially efficient and low-cost technology for determination of soil properties. Visible/near-infrared diffuse reflectance spectroscopy is a spectroscopic method that uses both the visible and near-infrared regions of the electromagnetic spectrum, in the range from 350–2500 nm. Many studies have shown that Vis-NIR combined with chemometrics is a rapid and objective method for quantifying several soil physical and bio-chemical properties [15–23]. In comparison with multi-spectral RS images, laboratory Vis-NIR produces a spectrum of much higher resolution (1–10 nm), signal-to-noise ratio, and atmospheric attenuation for the soil of a specific site. The key limitation of this technique is that laboratory Vis-NIR only provides spectral data at point scale. Hence, upscaling laboratory Vis-NIR spectra from point to landscape scale is essential to apply this technique to DSM. Many studies applied different techniques and environmental data for SOC spatial modeling [6, 13, 24–28]. For example, Zhang, Chen [26] used around 20 environmental data to estimate SOC contents and stocks at different depths in Denmark, the results could be used for future soil carbon assessment and inventories. In Denmark, over 60% of area is defined as agriculture land, covered by crops or by forest. However, this study did not apply any vegetation indices from RS data for SOC estimation. Thus, there might be some information from vegetation which was missed during the modeling process. Furthermore, no study has been conducted in geospatial modeling SOC across a larger landscape by combining laboratory spectrum and RS images. The first objective of this study in the Skjern river catchment, Denmark, was to combine Vis-NIR spectra and RS indices to improve predictions of topsoil SOC compared to using either one of the spectral data sources alone. Since, in general SOC content in wetland soils are higher than in upland soils, model stratification could be alternative way to improve model accuracy. Thus, the second objective was to improve the prediction of SOC by separately calibrating an upland model and a wetland model.

## Materials and Methods

### Study area

**Fig 1:**
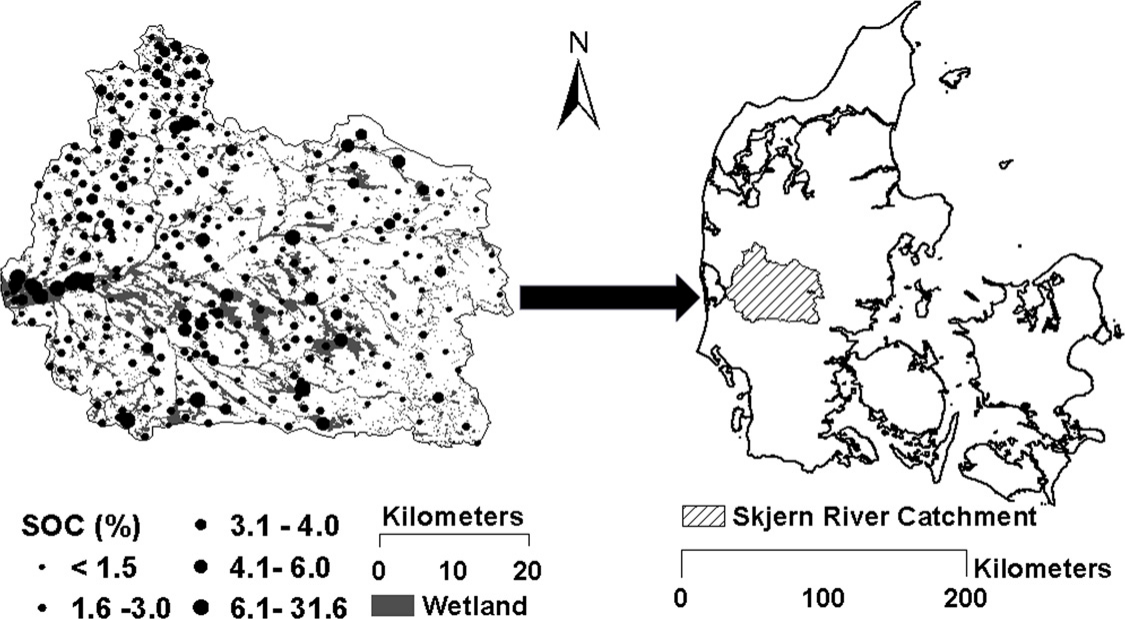
Study area of the Skjern river catchment and spatial distribution of measured soil organic carbon (SOC) in the topsoil (0 to 20 cm depth).

**Fig 2:**
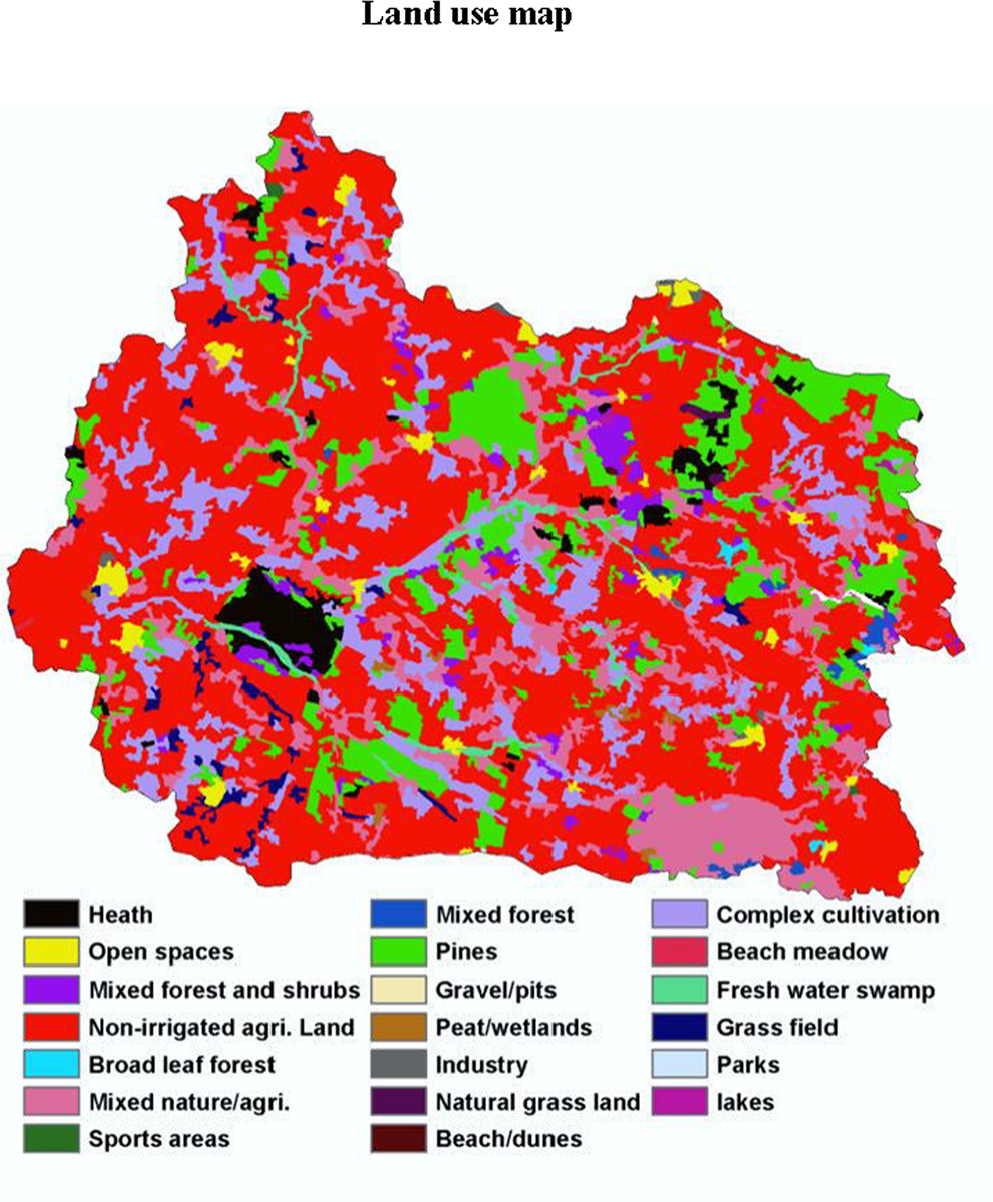
Land use map of the study area (Skjern river catchment) (20 classes).

The study area was the Skjern river catchment located in Western Jutland, Denmark (Fig 1), which covers an area of approximately 2,500 km^2^. This area was selected as a Hydrological Observatory (HOBE). The climate in this region is temperate maritime with a mean annual precipitation of 990 mm and mean annual temperature of 8.2°C [29]. The predominant soil texture is loamy sand [30]. The maximum elevation of the area is around 125 m above mean sea level. Over 50% of the land use is agriculture and this only on the upland area, and the main types of crop grown in this area are wheat and barley (Fig 2), followed by grass (30%), forests (7%), and heathland (5%) [29]. Approximately 19% of the entire area has been defined as wetland according to H. B. Madsen, A. B. Nørr [31] (Fig 1).

### Sampling and Laboratory analyses

A total of 328 topsoil (0–20 cm) samples were collected from the study area in the mid-1970s, 84 of these from wetland areas (Fig 1). All the sampling work was part of the Danish Soil Classification Database sampling. Danish agriculture ministry and local farmers issued permission for all the sampling work. The geographic position of the soil samples was marked with a pencil on a detailed topographic map. When returning to the office all sample locations were digitized into the GIS system. All the samples were originally from the Danish Soil Classification Database [30], which provides an SOC value for each sample. Each sample was composed of 25 subsamples which were taken within a 70 × 70 m square representing the specific soil characteristics of each site. Soil organic carbon was determined by combustion in a LECO IR-12 furnace [32]. Soil texture was analyzed using hydrometer and wet-sieving methods and only applied for samples with SOC values lower than 6%, resulting in 296 samples with soil texture values (Table 1).

**Table 1:** Descriptive statistics of soil organic carbon concentration in the topsoil of the Skjern river catchment, Denmark.

Property	Min	Max	Mean	SD	Median	Skewness	Cof.V	Kurtosis
SOC % (n = 328)	0.7	31.6	3.7	4.1	2.5	3.9	1.1	17.1

SD, standard deviation; n, number of samples; Cof.V, coefficient of variance.

### Laboratory spectra and RS data

Laboratory Vis-NIR spectra were obtained with a NIRSTM 2500 instrument (FOSS, Hillerød, Denmark). The spectral range was between 400 and 2,500 nm with a 0.5 nm resolution, giving 4200 spectral values. The instrument was calibrated every day before scanning by using NIRS DS2500 IU software (FOSS), and an artificial check sample as white reference was scanned every day before sample scanning. All the soil samples were air-dried before scanning to remove moisture effects, and the soil laboratory Vis-NIR spectra were obtained using the following method: A rotating sample cup (diameter: 7 cm) containing around 50 g of soil was scanned four times at seven different points and averaged. In total, 328 soil laboratory Vis-NIR spectra (absorbance value) were collected, and no outlier spectrum was detected based on principal component analysis. The instrument provided a high signal-to-noise ratio, and all spectra from the full 400–2500 nm range were used for further data analysis.

The RS data were extracted from the Satellite Pour l’Observation de la Terre (SPOT5) and Landsat Data Continuity Mission (Landsat8) images. All images were projected using ETRS89 map projection. Due to less cloud cover in summer, SPOT5 images for this study were obtained from June 2012 and Landsat8 images were taken from June and July 2013. The SPOT5 images were provided by the Danish Geodata Agency, with Band1 (500–590 nm), Band2 (610–682 nm) and Band3 (780–890 nm) having a 10-m spatial resolution, and Band4 (shortwave-infrared (SWIR); 1,580–1,750 nm) a 20-m spatial resolution. The Landsat8 images with spatial resolutions of 30 m, which included Band1 (blue 450–510 nm), Band2 (green 530–590 nm), Band3 (red 640–670 nm), Band4 (NIR 850–880 nm), Band5 (SWIR1 1570–1650 nm) and Band6 (SWIR2 2100–2290 nm) were provided by the U.S. Geological Survey (USGS2014). An atmospheric correction (including geometric and radiometric) was performed for all the images; the details of image correction are described by Guzinski, Nieto [33]. All the SPOT5 images were resampled to 30-m resolution using ‘bilinear resampling’ in ArcGIS.

In order to enhance spectral information and improve predictive capability for SOC, several spectral indices were derived from the satellite images (Table 2) following the approach by [9]. The following spectral vegetation indices were derived for both satellite images depending on their spectral bands: (i) from the Landsat8 image–Mid-Infrared Index (MidIR), Moisture Stress Index (MSI)[34], Normalized Difference Vegetation Index (NDVI), Normalized Difference Vegetation Green Index (NDVI green), Normalized Difference Water Index (NDWI)[35], Reduced Simple Ratio (RSR)[36], Simple Ratio (SR), Transformed Vegetation Index (TVI), Enhanced Vegetation Index (EVI)[37], Land Surface Temperature (LST), Emissivity (Emiss) and Leaf Area Index (LAI); (ii) from the SPOT image–MSI, NDVI, NDVI green, NDWI, RSR, SR, EVI and TVI. The formulas used to derive indices are shown in Table 2. The LAI was derived using decision-tree regression trained with high-quality MODIS LAI observations and Landsat reflectance, from all the VIS and NIR bands, affregated to MODIS pixel size [38]. Emiss was linearly scaled with fractional vegetation cover obtained from NDVI [39]. The LST was estimated using upwelling atmospheric radiance and atmospheric transmittance obtained from a MODTRAN run with the simulated sensor at satellite height following the approach of Coll, Galve [40]. The details of estimation of LST, Emiss and LAI are described in Guzinski, Nieto [33]. ArcGIS 10.2 was used to extract all the spectral indices and reflectance values for the sampling locations from the images and relate the extracted values to the corresponding SOC values.

**Table 2:** Description of vegetation indices.

Indices^a^	Equations	References
**NDVI**	$\frac{\text{NIR}\;-\;\text{Red}}{\text{NIR}\;+\;\text{Red}}$	[8]
**MSI**	$\frac{\text{MidIR}}{\text{NIR}}$	[34]
**NDWI**	$\frac{\text{NIR}\;-\;\text{SWIR}}{\text{NIR}\;+\;\text{SWIR}}$	[35]
**RSR**	$\frac{\text{NIR}}{\text{Red}}(1-\frac{\text{SWIR}\;-\;{\text{SWIR}}_{\text{min}}}{{\text{SWIR}}_{\text{max}}\;-\;{\text{SWIR}}_{\text{min}}})$	[36]
**TVI**	${\left(\frac{\text{NIR}\;-\;\text{Red}}{\text{NIR}\;+\;\text{Red}}+0.5\right)}^{1/2}\times 100$	[10]
**SR**	$\frac{\text{NIR}}{\text{RED}}$	[11]
**EVI** ^**b**^	$\text{G}\frac{\text{NIR}\;-\;\text{Red}}{\text{NIR}\;+\;{\text{C}}_{1}\text{Red}\;-\;{\text{C}}_{2}\text{Blue}\;+\;\text{L}}(1+\text{L})$	[37]
**NDVI Green**	$\frac{\text{NIR}\;-\;\text{Green}}{\text{NIR}\;+\;\text{Green}}$	[12]

^a^ NDVI, normalized differential vegetation index; EVI, Enhanced vegetation Index; MSI, Moisture Stress Index; NDWI, normalized difference water index; MSI: moisture stress index; NDWI: normalized difference water index; RSR: reduced simple ratio; SR: Simple Ratio; TVI: Transformed Vegetation Index

^b^ Empirical parameters for EVI: C_1_ = 6; C_2_ = 7.5; G = 2.5; L = 1.

### Ancillary environmental data

This study used plant-available water (PAW), elevation, soil maps, geology, land use, landscape type maps and remote sensing vegetation indices as predictors of SOC in the selected catchment area in Denmark. The PAW was considered to be one of the most important environmental factors for SOC, as also observed in other studies in relatively flat undulating landscapes with a large proportion of hydric soils and wetlands [24, 27]. The Danish national PAW map was derived from a Danish soil property map [30] via a pedotransfer function, the detailed calculation of which is described in Chapter 4 of the Atlas over Denmark [31]. The elevation of the soil surface was derived from light detection and ranging (LiDAR) technology where the LiDAR points were interpolated using the triangulation method and a fine-resolution digital elevation model (DEM) (grid spacing 1.6 x 1.6 m) generated by the National Survey and Cadastre of the Danish Ministry of Environment in 2011. The DEM was resampled to a grid size of 30 m using simple mean aggregation. Before resampling, the DEM was processed to remove the artificial sinks and peaks of 50 cm to ensure proper delineation of drainage networks.

Two soil maps, namely a soil class map and a soil texture map, were used. The soil class map consists of major FAO-UNESCO soil groups in the study area derived by Adhikari, Minasny [41] using decision tree analysis. The texture map was extracted from the Danish Soil Classification where texture information from the topsoil (0–20 cm) was used. The geology map provided information on the geological origin of soil material at 1 m depth, whereas the landscape map identified different landform types such as terminal moraine or glacio-fluvial plains, etc., in Denmark based on the geo-morphological features. The land use and land cover map used in this study was based on CORINE2000 data adjusted for Denmark [42]. Areas covered with forest, crops, grass meadows, etc., could be extracted from this map. The last three maps were originally vector-based and were rasterized to a grid resolution of 30 m for use in this study. More details of these ancillary environmental data can be found in Adhikari, Kheir [43].

### Regression modelling

All the models were developed using Cubist regression, and executed using the R-3.15.3 for windows statistical software (https://cran.r-project.org/bin/windows/base/old/2.15.3/) with the Cubist (Version: 0.0.18) package [44].

Cubist is a powerful data-mining tool for generating rule-based predictive models from data and has been developed from an earlier version of a C4.5 and M5 model tree [45]. A tree is grown where the terminal leaves contain linear regression models. There are also intermediate linear models at each step of the tree. All the models are based on the variables used in previous splits. A prediction is made using a linear regression model at each terminal node of the tree. The tree is finally reduced to a set of rules that initially are paths from the top of the tree to the bottom, and the linear model is then adjusted and simplified to reduce the absolute error [46, 47]. Generally, a Cubist regression model has a good predictive power and is also easy to understand and interpret [47]. This data-mining tool can also apply boosting-like scheme called committees where iterative model trees are created in sequence. The details of the boosting procedure can be found in Quinlan [46]. Furthermore, the Cubist model also provides the attribute usage (relative importance) of each variable which indicates the importance of the variable in the model. In the present study, we applied the “trainControl” function (R package: Caret (Version: 6.0–52) [48]) with 10 times iteration of 10-fold cross-validation to find the optimal number of committees, where the final decision regarding which model to use was based on the lowest root mean square error of cross-validation. All the R codes and packages are freely available in the internet.

The coefficient of determination (R^2^), R^2^ is the SSE (variance of the model's predictions)/SST (total variance), root mean square error (RMSE), and the RMSE value was calculated according to Eq 1. The ratio of performance to deviation (RPD) and the ratio of performance to interquartile distance (RPIQ) were used for assessment of model performance [49, 50]. The RPD is calculated as RPD = SD/ RMSE, The RPIQ was defined as RPIQ = IQ/SEP, where IQ = Q3-Q1; IQ being the interquartile distance of the validation set, Q1 the median of the first half of the validation set and Q3 the median for the second half of the validation set. All the statistic results presented in this paper only represent independent validation results.

$$RMSE=\sqrt{\frac{\sum {\left({\hat{y}}_{i}-y_{i}\right)}^{2}}{n}}$$ (Equation 1)

$\hat{\text{y}}$ is predicted value, y is observed value, n is the number of the samples.

### Identification of lab-spectral data to predict SOC and upscaling to the catchment scale

A Cubist model was developed from the Vis-NIR laboratory spectra and SOC concentrations using the whole dataset (N: 328 samples). The most important laboratory spectral wavelength/feature was selected based on variable importance ranking. In this paper, we only show the results from the most important laboratory spectral wavelength since inclusion of additional well-performing spectral lab wavelengths in the Cubist model did not lead to any significant improvement in SOC predictions. Afterwards, a kriging map based on this important spectral wavelength/feature was generated using (Empirical) Bayesian kriging (ArcGIS 10.2) [51]. Empirical Bayesian kriging is a geostatistical interpolation method, it also differs from classical kriging methods by automatically calculating parameters in order to achieve accurate results through a process of subsetting and simulations. Instead of using weighted least squares to estimate semivariogram parameters in ordinary kriging, this method uses restricted maximum likelihood. In general, the Standard errors of prediction from this method are more accurate than other kriging methods. In this work, default settings were chosen for the parameter of subset size and number of simulations (subset size = 100, number of simulations = 100), because there was no significant improvement in prediction results by adjusting these two parameters. Independent validation was applied for kriging map.

**Fig 3:**
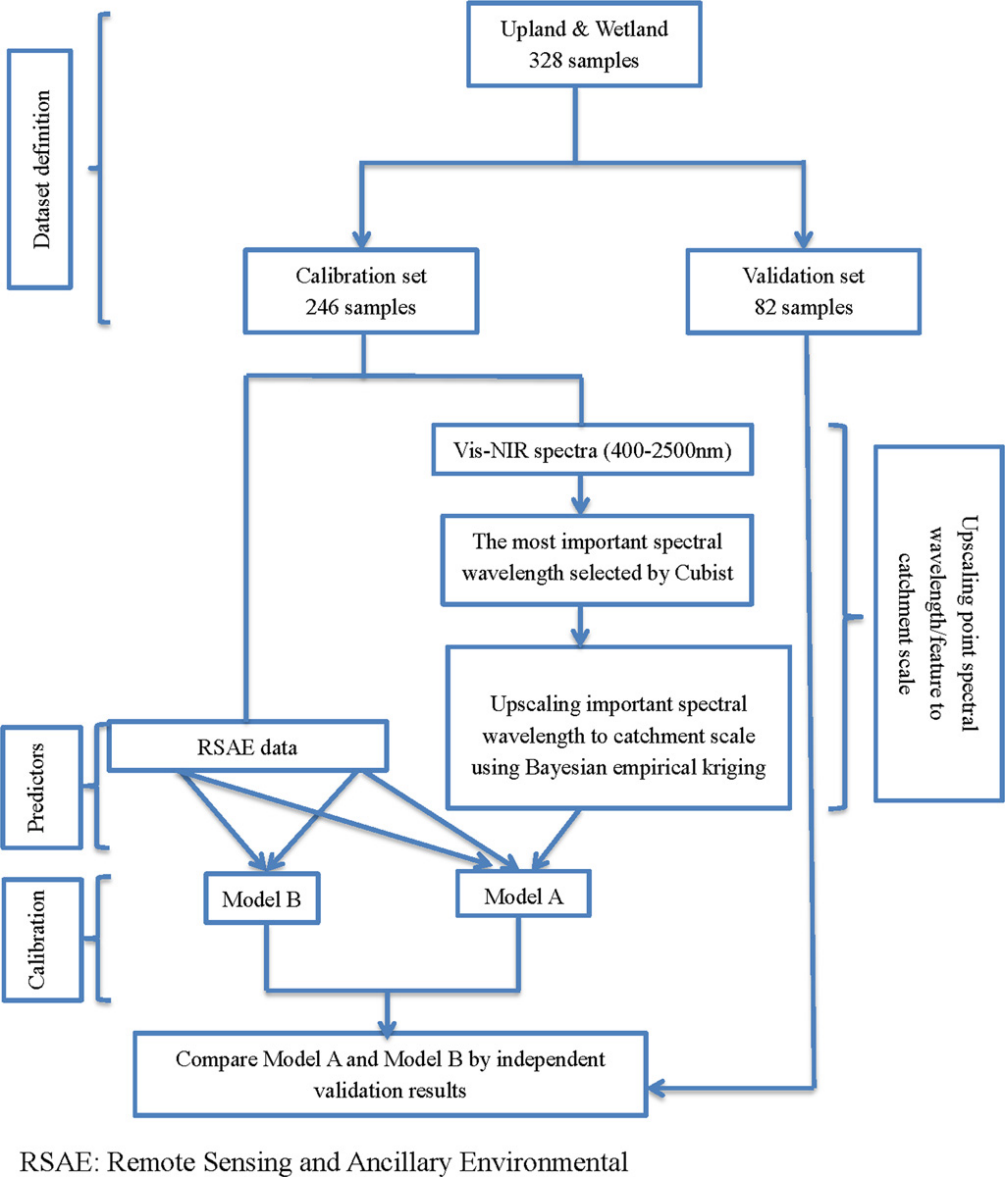
Flow chart summarizing the data integration process and model approach to upscale soil organic carbon across the study area, contrasting two distinct models (Model A and Model B).

This map (hereafter referred to as “estimated spectral map”) was converted to a raster with a 30-m resolution, and estimated spectral values were subsequently extracted from all 328 sampling points for further data analysis. The details of the upscaling procedure can be found in the flow chart in Fig 3.

In the spatial modeling step, for each model (A to D) we randomly selected 75% of the samples for calibration, withholding the remaining 25% samples for independent validation (Fig 1 & Table 2). In summary four models were generated:

Model A (UW): Developed from RS and ancillary environmental (RSAE) data and estimated lab spectral data. The model was derived from a total of 328 samples including both upland (U) and wetland (W) sites (calibration set: 246 samples, validation set: 82 samples);

Model B (UW): Developed from RSAE data alone and based on a total of 328 samples including both upland and wetland sites (calibration set: 246 samples, validation set: 82 samples);

Model C (U): Developed from RSAE and estimated lab spectral data based on a total of 244 samples confined to upland sites (calibration set: 183 samples, validation set: 61 samples).

Model D (W): Developed from RSAE and estimated lab spectral data based on a total of 84 samples confined to wetland sites (calibration set: 63 samples, validation set: 21 samples).

Two predictive maps with 30-m resolution were generated using a combination of models. The first map was generated using the combined ‘upland and wetland model’ (Model A (UW)) based on the RSAE data and estimated spectral map. The other was generated from combining models C and D. The predictive maps were produced using R with packages raster [52] and rgdal [53].

## Results

### Exploratory data analysis and laboratory spectral wavelength selection

The general statistics of SOC in the topsoil in the study area of the Skjern river catchment are shown in Table 1. The SOC content in the entire catchment ranged from 0.7% to 31.6%, showing that the study area was heterogeneous with large variations in SOC. The SOC statistics from different datasets are shown in Table 3. All these datasets include calibration and validation datasets for upland & wetland (UW) areas, wetlands (W) and uplands (U). In general, for each dataset the calibration set had a similar data distribution as the validation set. The range of SOC in all three calibration sets was wider than in the validation sets. The high SOC values were mainly from the W datasets, where the calibration and validation values were about three times higher than in the U datasets (Table 2). Generally, wetland soils had high SOC contents with a mean of 7.5% for the calibration sets and 6.3% for the validation sets. On the other hand, the boundary of the wetland was originally delineated in the 1920s. Since then it is likely that the SOC content declined in the wetland areas with a thin peat layer due to drainage and/or plowing in areas converted to agriculture [31]. Thus, in this study some samples classified as “wetland” showed low SOC concentrations of about 1%. A few samples located at the outlet of the catchment showed very high organic contents (Fig 1). This is because human activities have strongly affected part of this area, where the lower part of the Skjern River had been channelized and river valley wetlands were reclaimed for agricultural purposes from the 1960s until the beginning of this century [54]. Thus, some of the SOC had been lost from the soils in the carbon cycle, such as uptake by plants and carbon oxidizing back into the atmosphere.

**Table 3:** Descriptive statistics of soil organic carbon (SOC) from different datasets.

Datasets	Min	Max	Mean	SD	Median
		SOC (%)			
**Upland & wetland calibration set (n = 246)**	0.7	31.6	3.5	3.9	2.5
**Upland & wetland validation set (n = 82)**	0.8	26	4.2	4.6	2.6
**Upland calibration set (n = 183)**	0.7	5.5	2.5	0.9	2.4
**Upland validation set (n = 61)**	0.8	5	2.3	1	2.3
**Wetland calibration set (n = 63)**	1	31.6	7.5	7.3	3.7
**Wetland validation set (n = 21)**	1.8	20	6.3	5.1	3.2

SD, standard deviation; n, number of samples.

**Fig 4:**
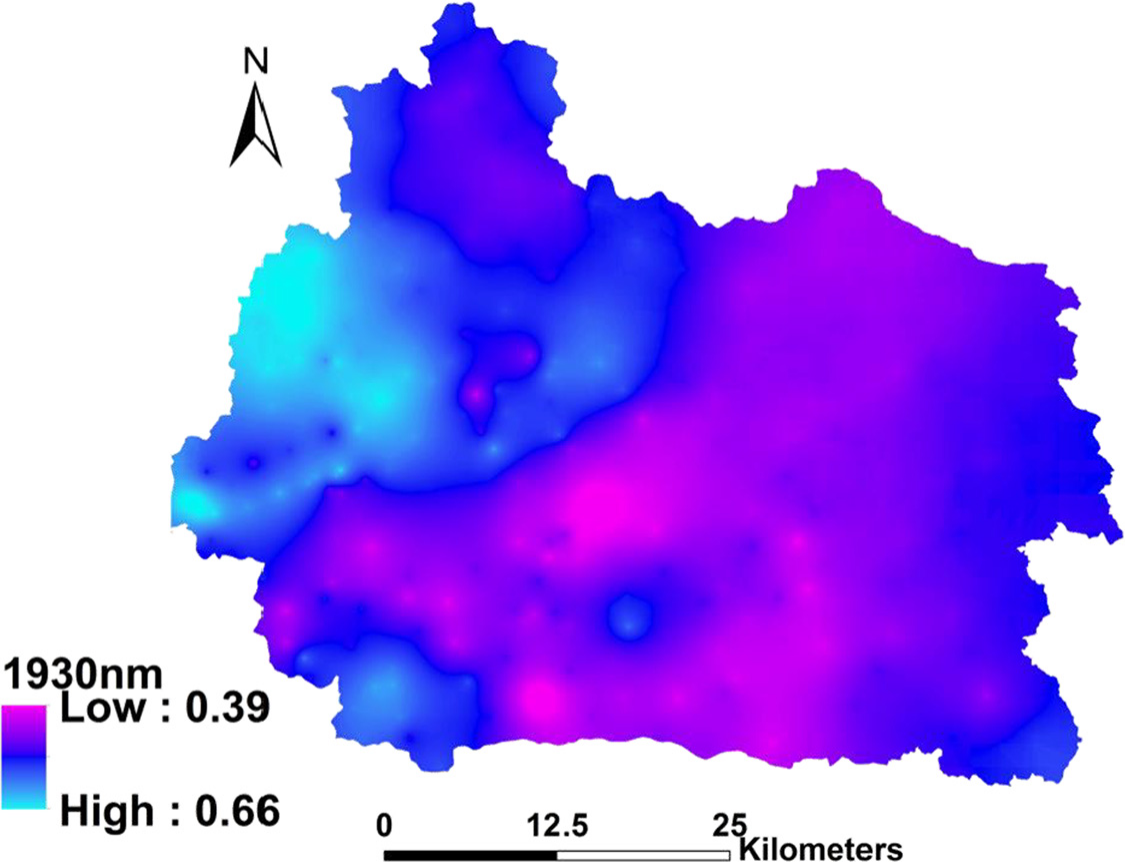
The NIR spectral feature (1930 nm) kriging map.

The wavelength of 1930 nm was selected as the most important laboratory spectral wavelength/feature based on the Cubist model. This spectral wavelength showed the highest attribute usage of 100%, which means that all the sub-models used this variable during the calibration process. It was also strongly associated with SOC content. The Cubist model also identified other wavelengths with more than 95% attribute usage, such as 628, 823, 1174, 2065 and 2309 nm. Since only one spectral wavelength (1930 nm) was involved in the spatial modeling process, we have focused on 1930 nm in this paper. Therefore, 1930 nm was selected as the most important wavelength in this study. The NIR spectral feature (1930 nm) kriging map is shown in Fig 4; the validation results showed an RMSE value of 0.04%.

### Comparison of Model Prediction

**Fig 5:**
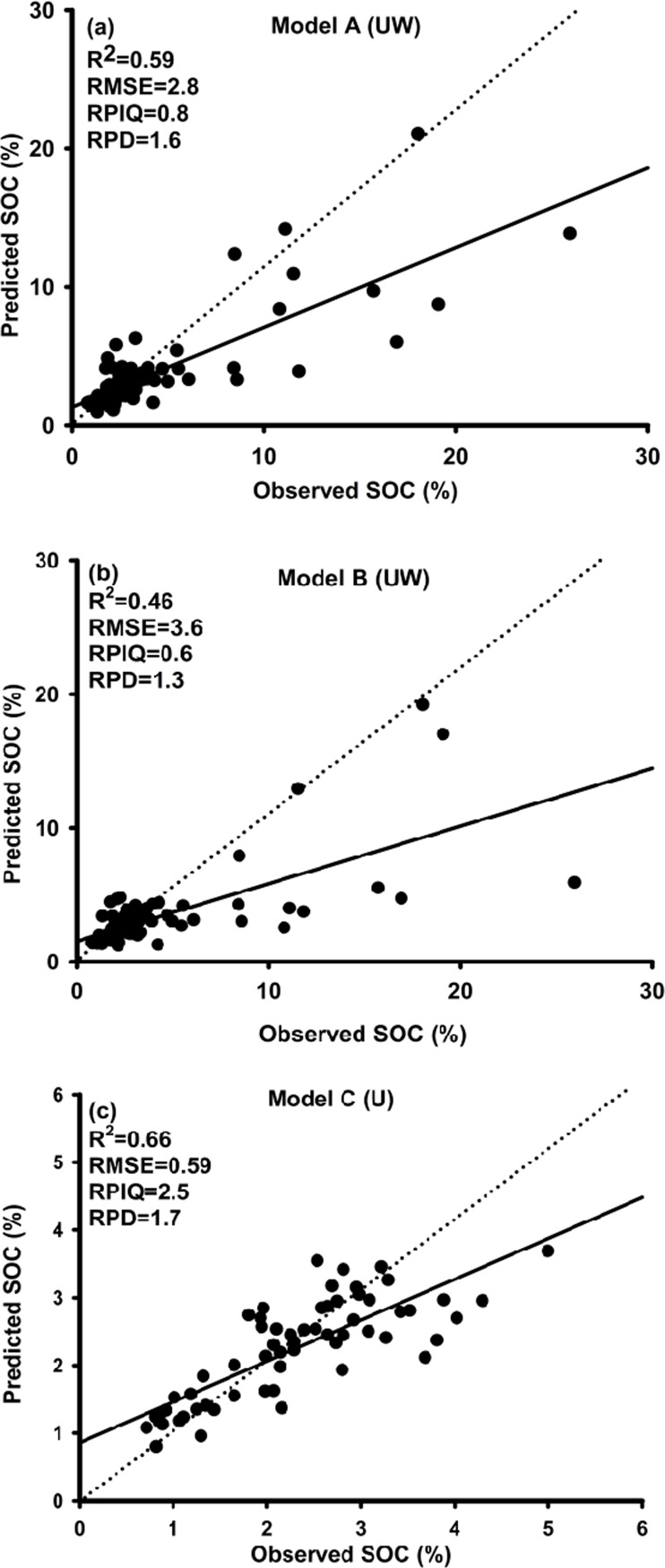
Independent validation results: Predicted vs. measured topsoil organic carbon concentrations using the Cubist model with different predictor datasets: (a) Prediction results from Model A (UW). The RSAE data and one estimated spectrum (1930 nm) were used for model calibration; the model was built on the combined upland & wetland dataset (validation: 82 samples). (b) Prediction results from Model B (UW). Only RSAE data were used for model calibration, and the model was based on the same soil dataset as model A (UW). (c) Prediction results from Model C (U). The RSAE data and one estimated spectrum (1930 nm) were used for model calibration; the model was built on only the upland soil dataset (validation: 61 samples).

The observed versus predicted values for Model A and Model B are shown in Fig 5A and 5B, respectively. These two models were based on the same calibration and validation samples from upland and wetland sites, but developed by different predictors. Model A differed from Model B. The Model A including one additional predictor–the geospatial estimates of the 1930 nm spectral data derived from Bayesian kriging. Model A that used the estimated spectral map produced better prediction results with values of R^2^, RMSE, RPIQ and RPD of 0.59, 2.8%, 0.8 and 1.6, respectively, vs. 0.46, 3.6%, 0.6 and 1.3 for Model B. These findings suggest that the Vis-NIR spectral estimates improved the SOC predictions (Fig 5A and 5B). This further implies that the point-specific Vis-NIR spectral data had the potential to improve geospatial predictions of SOC across the whole catchment, despite the uncertainty in 1930 nm spectral estimates derived from the interpolation using Bayesian kriging.

As mentioned in an earlier section, the Cubist model also identified a few more spectral variables (e.g. 825 nm, 1138 nm, 1754 nm and 2033 nm) with attribute usage of more than 95%. However, their use in spatial modeling did not significantly improve the results after adding the 1930 nm wavelength. This may be explained by uncertainties in the NIR spectral feature map. Since this map was estimated by kriging, the independent validation results showed that around 60% of the variance was explained in kriged map. This error was a factor to the spatial modeling process when we combined all the predictors. In essence, adding additional interpolated NIR spectral bands to the SOC model may introduce more errors rather than aiding SOC predictions. Therefore we only included one specific spectral variable (1930 nm) in the spatial modeling process. Finally, Model A (UW) was developed from 28 predictors, which included just the one estimated spectral map (1930 nm) and 27 RSAE predictors. The details of these predictors can be found in Table 4 and will be discussed in a later section.

**Table 4:** List of environmental variables, vegetation index derived from remote sensing images and one estimated spectrum (1930 nm) used to predict the distribution of soil organic carbon in the Skjern river catchment.

Environmental variables	Type of variable	Description	Range of values	Scale or resolution	Mean	Median	SD
**Soil texture map**	Categorical	Map of Soil types based on soil texture (8 classes)	−	1:50,000	−	−	−
**Soil class map**	Categorical	FAO–UNESCO soil groups(10 classes)	−	30 m	−	−	−
**Geology**	Categorical	Scanned and registered geological map (48 classes)	−	1:100,000	−	−	−
**Land use**	Categorical	CORINE Land cover data adopted in Denmark (20 classes)	−	1:100,000	−	−	−
**Landscape type**	Categorical	Landform types (8 classes)	−	1:100,000	−	−	−
**Elevation (m)**	Continuous	Elevation of land surface derived from LiDAR (m)	0–137	30 m	48.5	45.6	24.7
**Plant available water**	Continuous	The plant available water content in the root zone (vol. %)	7.7–35.3	30m	16.5	15.6	3.47
**SOPT5 NDVI (June/2012)**	Continuous	Normalized Difference Vegetation Index from SPOT5	-0.40–0.57	30 m	0.07	0.08	0.17
**Landsat NDVI (June/2013)**	Continuous	Normalized Difference Vegetation Index from Landsat 8 June	-0.97–0.92	30 m	0.42	0.4	0.18
**Landsat NDVI (July/2013)**	Continuous	Normalized Difference Vegetation Index from Landsat 8 July	-0.95–0.96	30 m	0.69	0.75	0.19
**1,930 nm**	Continuous	Estimated spectra feature in 1,930nm	0.41–0.64	30 m	0.52	0.52	0.04

Model A and Model C. Model C used similar predictors to Model A, but neither model was based on completely the same samples (Model A included both upland and wetland samples (328), Model C only the upland samples (244) that Model A also used). The prediction results from Model C are shown in Fig 5C. Model C resulted in an R^2^ of 0.66, RMSE of 0.59%, RPIQ of 2.5 and RPD of 1.7. Model A and Model C were based on samples with a different range of SOC. Especially the SOC contents in the validation set of Model A ranged from 0.8 to 26%, but the SOC in the validation set of Model C ranged from 0.8 to 5% (Table 3). Thus, it was not fair to directly compare the prediction accuracy indexes from both models. We therefore recalculated the RMSE value based on predicted values of only upland samples using Model A and the reference values, resulting in a value of 0.87%. Then we could directly use this value to compare the RMSE value of Model C (0.59%). The results showed that the RMSE value of Model C was still lower than that from Model A and that the RPIQ and RPD values from Model C were considerably better than results from Model A. This means that the predicted values of upland samples from Model A had lower prediction accuracy than from Model C. Based on this result, we concluded that the prediction accuracy of upland samples was improved by model stratification. This was because the variation in SOC in Model A samples was much higher than for Model C (Table 3), with respective ranges of 0.7‒ 31.5% and 0.7‒ 5.5%.

We also individually calibrated samples derived from wetland samples alone (Model D), but the results were not acceptable. The RMSE was very high (9.6%) and R^2^ close to zero (prediction results not shown). The reason why this model could not perform well might be explained by the vegetation coverage of the wetland area and the complexities of the wetland ecosystem. The wetland area in the Skjern catchment was dominated by perennial rye and reed canary grasses, and the vegetation coverage in this area was very uniform during the summer season. Therefore, the RS images probably could not give sufficient discrimination of vegetation using the vegetation indices derived from summer images. It is impossible to find cloud-free images during the winter season in Denmark.

### Prediction maps

**Fig 6:**
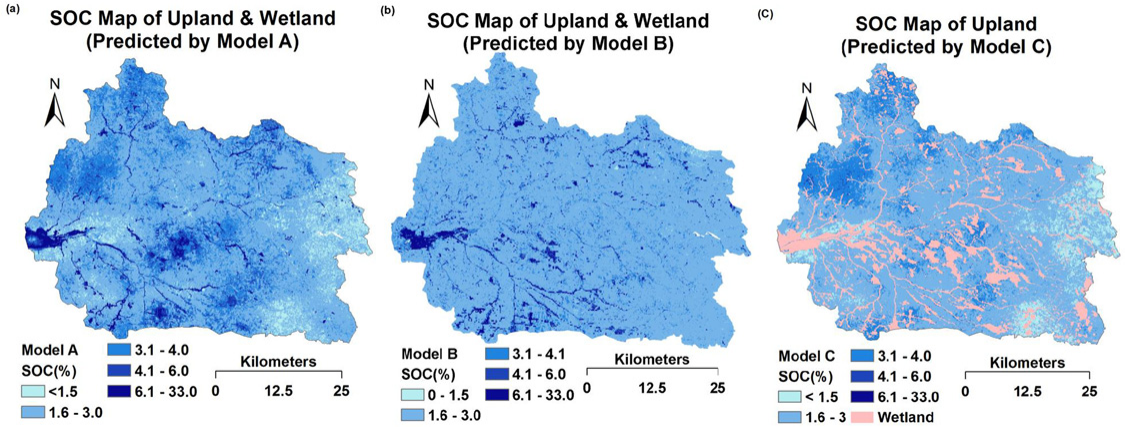
Prediction maps (30-m resolution) of topsoil organic carbon (SOC) using the Cubist model. (a) Map for upland and wetland, predicted by Model A based on ancillary environmental data, remote sensing data and the estimated spectrum (1930 nm). (b) Map for upland and wetland, predicted by Model B based on ancillary environmental data and remote sensing data. (c) Map for upland, predicted by Model C based on ancillary environmental data, remote sensing data and the estimated spectrum (1930 nm).

Three prediction maps of SOC are shown in Fig 6, where maps (a) and (b) are ‘upland & wetland’ maps, but predicted by Model A and Model B, respectively. The only difference between these models was that Model A included the NIR spectral feature (1930 nm) map and Model B did not. When comparing these two maps, map (a) produced a much more detailed and accurate picture based on visual inspection by experts than map (b). Firstly, map (a) accurately shows highly organic soils in the westernmost part of the catchment area, whereas map (b) shows a much lower SOC content. Secondly, in map (a) the northwestern, middle and southern parts of the catchment area have relatively high SOC contents, because the northwestern part was dominated by forest, and the middle and southern parts by dense wetlands. Lastly, in map (a) the eastern part of the catchment had relatively low SOC contents, which might be explained by the geological history. In this area, the soil was dominated by aeolian deposits, mainly with coarse sand; most of the organic matter and clay was removed by wind erosion during the 16th and 18th centuries [31]. These details were not all clear in map (b). This could be why Model B produced a much higher RMSE than Model A. This significant difference between the maps could also be explained by the NIR spectral feature (1930 nm) map (Fig 4), where this single variable provided very important and much more detailed information on SOC for Model A. If we compare this kriged map (Fig 4) with map (a) (Fig 6), we can find some similar spatial distributions. In the kriged map, the outlet and the northwestern part of the catchment showed a high absorbance value for wavelength 1930 nm, which indicates that the soils here contain more carboxylic acids (C = O) than the rest of the area.

The SOC for upland areas was predicted by Model C (Fig 6C). Both Model A and Model C included RSEA data and the estimated spectra (1930 nm), but had different numbers of soil samples. In general, these two maps showed similar spatial distribution patterns of SOC. However, map (a) generally showed slightly higher SOC values compared with map (c) because Model A included some highly organic soil samples from the wetland area. Consequently, Model A predicted relatively higher SOC values compared with Model C. This could explain why Model C gave lower prediction errors than Model A because Model C was based solely on upland samples with a substantially lower range and coefficient of variation of SOC concentrations.

### Spatial modeling variable selection

The final values used for the ‘upland & wetland’ Cubist model (Model A) and ‘upland Cubist model’ (Model C) were 20 and 10 committees, respectively. The soil class map (FAO-UNESCO soil groups) and land use map (CORINE2000 data) were selected to set the initial rule conditions for most of the rules. For instance, the soil class map was used for rule setting in 46% of the models. The Cubist regression tree produces linear equations for SOC predictions based on different rules. Here we show an example of the rule used for SOC predictions in Model A:

Rule 1: [237 cases, SOC (in %) mean 3.29, range 0.65 to 31.56, est err 1.33]

If Soil class map in (2, 4, 5, 6, 7, 8) (The numbers indicate different soil classes) PAW > 14.30 then SOC = 13.6151138–0.3815 LST (June) + 0.3723 LST (July) - 3.315 NDVI (June) + 3.166 NDVI (July)– 1.28 SR (July) + 6.92 NDWI (June) + 0.369 PAW + 2.54 SR (June) + 13.9 1930nm—3.39 NDVIgreen (July) + 2.24 EVI (July) + 1.92 NDVIgreen (June)

From the model-building process, we can see that the Cubist model did not select all the predictors for use in the prediction. For example, none of the raw spectral band from any of images was selected by Cubist model. For the linear model building, the estimated spectral map, PAW, the vegetation indices NDVI, LST, EVI, etc., provided very high attribute usage. Table 5 shows the top 10 predictors selected by the Cubist model and their attribute usage. The predictor PAW had 100% attribute usage. The second-highest ranking predictor of SOC was the ‘estimated spectral map’. The Pearson correlation coefficient value between the PAW and SOC was 0.17, and the correlation coefficient value between the ‘estimated spectral map’ and SOC was 0.15 ‒ the highest correlation coefficients of all the predictors. In this study, the catchment area was dominated by non-irrigated agricultural land and was mainly covered by crops. Therefore, different vegetation indices were applied to accentuate specific vegetation properties. Vegetation indices such as NDVI, EVI, and LST also showed high attribute usage in this study, consistent with previous soil studies [1, 3, 7, 55]. As shown in Table 5, vegetation indices from Landsat8 had better predictive ability than the indices derived from SPOT5. The NDVI values derived from Landsat8 had a much wider range and higher mean and median values and wider ranges than those derived from SPOT5 (Table 4).

**Table 5:** Top 10 predictors selected by the Cubist calibration model A, B, C and their attribute usage ranking.

Model A	Attribute usage	Model B	Attribute usage	Model C	Attribute usage
**PAW (%)**	100 %	PAW	93%	PAW	100%
**1,930 nm**	96%	Landsat8 NDVI(July)	82%	1,930nm	98%
**Landsat8 NDVI(July)**	88%	Landsat8 NDVI(June)	77%	Landsat8 NDVI(July)	90%
**Landsat8 NDVI(June)**	85%	Landsat8 EVI(July)	70%	Landsat8 NDVI(June)	87%
**Landsat8 EVI(July)**	80%	Landsat8 SR(July)	63%	Landsat8 LST(July)	84%
**Landsat8 EVI(June)**	68%	Landsat8 NDVIgreen (July)	52%	Landsat8 LST(July)	75%
**Landsat8 SR(July)**	56%	Landsat8 EVI(June)	48%	Landsat8 NDVIgreen (July)	68%
**Landsat8 NDVIgreen_July**	40%	Landsat8 SR(June)	41%	Landsat8 NDVIgreen (June)	61%
**SPOT5 SR**	38%	SPOT5 NDVI	33%	SPOT5 SR	44%
**SPOT5 NDVI**	29%	SPOT 5 SR	26%	Elevation	22%

PAW: plant available water; NDVI: normalized differential vegetation index; EVI: Enhanced vegetation Index; SR: Simple Ratio

Model A, upland and wetland model based on ancillary environmental data, remote sensing data and the estimated spectra (1930 nm)

Model B, upland and wetland model based on ancillary environmental data and remote sensing data

Model C, upland model based on ancillary environmental data, remote sensing data and the estimated spectra (1930 nm).

Interestingly, we found a high attribute usage of LST for the ‘upland model’ (Model C), but not for the ‘upland & wetland model’ (Model A). The LST estimates surface energy fluxes that are more closely related to the physiological activities of leaves in a vegetated area [56]. Upland ecosystems are much more diverse than wetlands in terms of vegetation and land use that both may impact energy fluxes. NIR light energy is preferentially absorbed by water in wetlands. Therefore, upland ecosystems release electromagnetic energy back to the atmosphere much faster than wetlands [57], suggesting that the LST index is more sensitive for upland than wetland ecosystems.

## Discussion

### Laboratory spectra interpretation

In general, the absorption in the NIR region between 1000 and 2500 nm can be attributed to water, clay minerals and organic matter mainly composed of SOC. Organic molecule overtones and combination bands occur in the NIR region due to stretching and bending of the NH, CH and CO groups ‒ a result of the organic matter content [49, 58]. The main process by which molecules absorb energy is electronic transitions in atoms from ground to higher energy states. Previous research has shown that bands around 1100, 1600, 1700–1800, 1900, 2000, and 2200–2400 nm are important for SOC calibration [14, 50, 59–65]. Rossel, Walvoort [66] also found a strong absorption peak around 1930 nm that is associated with organic compounds such as organic acids and alkyls. Our result confirms, as also concluded by Rossel and Behrens [64], that 1930 nm is highly related to carboxylic acids (RCOOH). Carboxylic acids exist as dimers because of strong intermolecular hydrogen bonding. Thus, the O-H stretching band, commonly attributed to free water, can also be found around 1930 nm. Base on the information of The Danish Soil Classification: Atlas over Denmark[31], most soils in the study area were sandy with over 90% of sand content. In this type of soil, soil moisture is mostly held by soil organic matter that is highly correlated to SOC rather than to clay.

### Spatial predictions of SOC

The Model A produced better prediction results than Model B, due to estimated spectral map involved in Model A. This might be explained by the fundamentals of laboratory spectroscopy, which provided spectral information directly linked to SOC. Knox, Grunwald [67] explained as much as 85% (Vis-NIR) and 96% (mid-infrared) of the variation in various soil carbon pools. They found that the spectral region above 2000 nm contributed most to carbon fraction models (SOC, recalcitrant carbon, hydrolyzable carbon, and total carbon) based on a large dataset with a wide range in soil C values and more than 1000 samples.

Moreover, all soil samples used in the study presented here were composed of 25 subsamples which were taken within a 70 × 70 m area, suggesting that soil samples were representative of the soil properties of the sites. Furthermore, the SOC map was predicted with a 30-m resolution. Each soil laboratory spectrum thus provided soil property information not only for a single sampling point, but also for the neighboring area. This block-based sampling is useful for building soil prediction models that incorporate RS images, specifically those with 30-m resolution as used in this study.

Studies that incorporate Vis-NIR data in the modeling process to make spatial predictions across a landscape are still rare. For example, Sarkhot, Grunwald [68] predicted hot-water-extractable soil carbon (HC) using site-specific lab-measured HC and kriging (model 1) and Vis-NIR-estimated HC derived from Partial Least Square Regression that were upscaled using kriging to a floodplain area in Texas, U.S. (model 2). The fusion of lab and spectral soil data using a variety of quantitative methods is becoming more popular because of the ability of the spectra to improve soil predictions (Grunwald et al., 2015). Our findings reveal that the strategy of using (Empirical) Bayesian kriging to upscale laboratory spectra from point to grid (raster) scale and combining those with RS images for geospatial modeling across a larger landscape yielded improved SOC predictions compared to traditional univariate SOC models.

### Spatial modeling variable importance

Our study revealed that the PAW is the most important variables for SOC predictions. No study has directly used PAW as a predictor to calibrate with SOC, but some studies show the potential of using different soil property maps as predictors for modeling SOC [24, 27]. Our results fell into that category, because the PAW map was derived from the Danish national soil property map, which was highly correlated with SOC content.

As mentioned in results section, due to the technical specifications and environmental conditions for Landsat8 and SPOT5, there are big differences in the reflectance values (Table 4). In this study, the RS images were acquired in two different years (SPOT5 in 2012 and Landsat8 in 2013), due to the need to get cloud-free images. Consequently, there may have been some differences in growing conditions between 2012 and 2013. Additionally, and most likely, all the SPOT5 images were resampled from 10-m to 30-m resolution using the ‘bilinear resampling’ function from ArcGIS. The bilinear resampling method performs a bilinear interpolation, the output new value of each cell based on the four nearest neighbor values, so it is recommended for continuous data. On the other hand, this resampling method may introduce new values never found in the original image and also some blurred edges (ESRI, 2014).Therefore, pixels with high digital number values from SPOT5 images were averaged during the resampling process.

For RS vegetation indices, all the satellite images used in this study were from June and July, which are the most productive months for crop growth. The NDVI is chlorophyll-sensitive and indicates the greenness of the plant canopy, which reflects crop growth characteristics and indirectly gives information on soil properties at specific sites. Therefore, this index has been used to infer vegetation productivity or biomass status [69–71]. We also found that NDVIgreen from June and July was a significant predictor for the ‘upland model’ (Model C), probably because NDVIgreen was developed mainly for upland vegetated areas [9]. The results also showed a strong attribute usage of EVI for the ‘upland & wetland model’ (Model A). The EVI is more responsive to canopy structural variations, including LAI, canopy type, plant physiognomy, and canopy architecture than NDVI (Huet et al., 2002). These two indices complement each other and extract canopy biophysical parameters [35, 72].

Since no raw RS spectral band selected by either Cubist model, which means that the RS vegetation indices are more suitable than raw spectral bands as indicators for crop and SOC. Vegetation indices derived from satellite images accentuate vegetation-specific properties and allow the inference of specific crop/vegetation/bare soil characteristics and are thus better suited than raw bands for soil modeling [73]. The RS images applied in this study were extracted from June and July, and the study area was mainly covered by crops, grass and forest that are discernible using vegetation indices. Under this condition, RS vegetation indices can be used for modeling purposes by RS systems to identify biophysical features measured on the Earth’s surface. Thus, the quantitative relationships between RS vegetation indices and SOC can be found by an empirical modeling approach.

### Use of RS indices for SOC predictions in wetland soils

Since RS indices indicate biophysical vegetation properties and productivity in vegetation, using RS images for soil spatial modeling indirectly extracts soil information from the vegetation cover. It means that an indirect statistical relationship can exist between RS indices and soil properties [7, 74]. However, in the present study, all wetland samples sites showed very similar values for RS vegetation indices in corresponding pixels. For instance, in the wetland validation sample set, the minimum and maximum values for SOC were 1.8% and 20%, respectively (Table 2). For the corresponding sampling sites, the pixel values of NDVI from June were 0.21 and 0.19. This is because the wetland area in the Skjern catchment was barely covered by vegetation and most of light energy was absorbed by water. This gave relatively homogenous distribution of NDVI value in the wetland area compared to NDVI values from the upland area. A similar situation, but only in the wetland area, was observed from other RS vegetation indices such as EVI and NDVI green. Therefore, in this situation, the linear relationship was not strong enough to make a robust model when we calibrated SOC and RS vegetation indices for wetland soils. Consequently, most of the validation samples with high SOC contents were underestimated in Model A and Model B (Fig 5A and 5B). In contrast, if we look at the validation set used to test Model C (U) (model based only on upland samples), this validation sample set shows minimum and maximum values of SOC of 0.8% and 5% (Table 3), respectively, and corresponding pixel values of NDVI of these two sites from June of 0.41 and 0.75. Here the Cubist regression tool is able to build a linear relationship between SOC and vegetation indices, because the higher pixel value of the NDVI indicates relatively higher SOC at the corresponding sampling sites.

Vis-NIR has limited ability to predict SOC in the high carbon range. This has been demonstrated in numerous studies [20–22, 59, 75, 76]. Since Model D (W) was based only on soils with relatively high SOC concentrations (up to 31.6% in the calibration and 20% in the validation set) compared with the upland sites (5.5% in the calibration and 5.5% in the validation set) (Table 3), the inclusion of the lab-based spectral map was unable to improve the predictive capability of Model D (W).

## Conclusions

The present study presents a novel approach for upscaling laboratory spectral wavelengths and features from point to regional scale and combining these with multi-spectral images to predict SOC. The results showed that the prediction accuracy was remarkably improved by adding one estimated laboratory spectral image when compared with a model developed from only RS data and ancillary environmental data. The modeling process also showed that vegetation indices, such as NDVI, EVI and NDVIgreen, were very important predictors for SOC spatial modeling. Findings suggest that laboratory hyperspectral data can be used as an alternative to RS images to model SOC, which potentially reduces cost and labor. Another drawback of freely available RS images is the large pixel size (10–30 m) and wide spectral bands. This study demonstrated that laboratory spectral data can be used not only for quantitative analysis of a single soil sample using chemometrics, but also to facilitate regionalization and improvement of the SOC prediction accuracy, specifically if combined with ancillary environmental data and/or RS data. Despite interpolation errors of SOC using Bayesian kriging, the incorporation of one spectral band (1930 nm) in the modeling process significantly improved the overall prediction accuracy and model fit for SOC in the topsoil.

For future use of this novel approach, there is potential in combining more data resources from different sensors for spatial modeling. Since predictions for most soil properties can be improved when based on a combination of data than based purely on either environmental data or individual sensors. In our study, we also found that separately calibrating wetland and upland datasets improved prediction results only for the upland set. The RS images were only available for the summer months, which limited discernment of vegetation-specific features in the Skjern river catchment.
